# SNP Identification by Transcriptome Sequencing and Candidate Gene-Based Association Analysis for Heat Tolerance in the Bay Scallop *Argopecten irradians*

**DOI:** 10.1371/journal.pone.0104960

**Published:** 2014-08-14

**Authors:** Xuedi Du, Li Li, Shoudu Zhang, Fei Meng, Guofan Zhang

**Affiliations:** 1 National & Local Joint Engineering Laboratory of Ecological Mariculture, Institute of Oceanology, Chinese Academy of Sciences, Qingdao, Shandong, China; 2 University of Chinese Academy of Sciences, Beijing, China; 3 Department of Information Analysis, Biomarker Technology Corporation, Beijing, China; The Ohio State University, United States of America

## Abstract

The northern bay scallop *Argopecten irradians irradians* (Lamarck) and the southern bay scallop *Argopecten irradians concentricus* (Say) were introduced into China in the 1980s and 1990s, and are now major aquaculture molluscs in China. Here, we report the transcriptome sequencing of the two subspecies and the subsequent association analysis on candidate gene on the trait of heat tolerance. In total, RNA from six tissues of 67 and 42 individuals of northern and southern bay scallops, respectively, were used and 55.5 and 34.9 million raw reads were generated, respectively. There were 82,267 unigenes produced in total, of which 32,595 were annotated. Altogether, 32,206 and 23,312 high-quality SNPs were identified for northern and southern bay scallops, respectively. For case-control analysis, two intercrossed populations were heat stress treated, and both heat-susceptible and heat-resistant individuals were collected. According to annotation and SNP allele frequency analysis, 476 unigenes were selected, and 399 pairs of primers were designed. Genotyping was conducted using the high-resolution melting method, and Fisher’s exact test was performed for allele frequency comparison between the heat-susceptible and heat-resistant groups. SNP all-53308-760 T/C showed a significant difference in allele frequency between the heat-susceptible and heat-resistant groups. Notably, considerable difference in allele frequency at this locus was also observed between the sequenced natural populations. These results suggest that SNP all-53308-760 T/C may be related to the heat tolerance of the bay scallop. Moreover, quantitative expression analysis revealed that the expression level of all-53308 was negatively correlated with heat tolerance of the bay scallop.

## Introduction

The bay scallop, *Argopecten irradians*, is naturally distributed along the eastern coast of the United States and the Gulf of Mexico, and it includes four subspecies: *A. i. irradians* (Lamarck), *A. i. concentricus* (Say), *A. i. amplicostatus* (Dall) and *A. i. taylorae* (Petuch). The northern bay scallop *A. i. irradians* was first introduced to China in 1982 [1], [2], and additional introductions were conducted in 1991 and 2002 from Canada and in 1998–1999 [3] from Virginia and Massachusetts. The southern bay scallop *A. i. concentricus* was introduced from Florida and North Carolina in 1991 [4] and 1995 [5], respectively. The bay scallop is currently one of the major aquaculture molluscs in China, with the northern bay scallop mainly cultured in the Bohai Sea and North Huanghai Sea and the southern bay scallop mainly cultured in Beibu Bay of China. Over the past few decades, researchers and breeders in China have been making great efforts to improve the growth vigor of the bay scallop through group breeding and successive selection in growth [6]–[9]. Two varieties, called “zhongkehong” (accession number: GS-01-004-2006) and “zhongke No. 2” (accession number: GS-01-005-2011), were cultivated with superior growth rates. However, summer mass mortality remains a great challenge in scallop aquaculture in North China and causes great economic losses each year, and heat stress is suspected to be one of the main environmental inducers [10], [11]. Thus, it is of great importance to explore the heat tolerance mechanism of the bay scallop, particularly under the context of global warming. Zheng et al. [12] reported that intercrossing between the two subspecies was feasible and described the heterosis in growth, which indicated that crossbreeding between northern and southern bay scallops would have great potential application value in bay scallop aquaculture. Given the huge difference in the sea surface temperature between their natural distribution range in America, as recorded by NOAA from 1990 to 2013 (http://www.esrl.noaa.gov/psd/data/gridded/data.noaa.oisst.v2.html), it is generally assumed that the northern and southern bay scallops differ genetically in heat tolerance. Thus, intercrossed individuals are desirable for the genetic dissection of the heat tolerance of the bay scallop.

Many studies suggest that heat shock proteins, antioxidant enzymes and the ubiquitin-proteasome system are very important for plants and animals to adapt to temperature change by keeping the proteins at homeostasis and eliminating reactive oxygen species (ROSs) [13]–[16]. In scallops, superoxide dismutase (SOD) activity and the mRNA expression of heat shock protein 70 (*HSP70*) increased substantially under high temperature exposure [17], [18], and the increased heat shock proteins were suspected to be related to the induced heat tolerance of the bay scallop [19], [20]. In the zhikong scallop (*Chlamys farreri* Jones et Preston), one SNP in *HSP22* associated with molecular chaperone activity and heat tolerance was revealed [21]. In addition, a variation in the promoter region affecting metallothionein gene expression was detected in bay scallops under heat stress [22]. However, SNPs from the limited number of genes could hardly provide a comprehensive view of the response and adaptation mechanisms to heat stress. Because the cost of next-generation sequencing has decreased dramatically, transcriptome sequencing using pooled RNA from multiple individuals is an effective approach for massive SNP identification in gene coding regions, and it has been applied in a number of species, including maize [23], oilseed rape [24], catfish [25], chum salmon [26] and black cottonwood [27].

Association analysis has become popular in the past several years for trait dissection and has yielded very promising results in human disease research [28]–[31] and agronomic character analysis [32]–[35]. In contrast to data-driven genome-wide association study, in which genotyping of thousands to millions of SNPs in hundreds to thousands of samples is required, hypothesis-driven candidate gene association analysis is directed at genes that have clear roles in controlling special traits and thus dramatically decreases the genotyping workload [36]–[38].

In this study, transcriptome sequencing was conducted for both northern and southern bay scallops, providing useful resources including unigene sequences and plentiful SNPs for further research. Moreover, hundreds of SNPs were validated, and a candidate gene association analysis was conducted in intercrossed materials with one SNP identified been closely related to the heat tolerance of the bay scallop. Quantitative expression analysis further proved that the expression level of all-53308 was negatively correlated with the heat tolerance of the bay scallop.

## Materials and Methods

### Ethics Statement

The bay scallop *Argopecten irradians* used in this study was a marine mollusc species and was brought from the farm and acclimated in the aquarium at the Institute of Oceanology, Chinese Academy of Sciences (IOCAS). All of the experiments were conducted according to local and national regulations. No specific permissions were required for the collection of bay scallops and the experiments described. All of the field studies were conducted at the culture station of IOCAS in Jiaonan, Qingdao and did not involve any endangered or protected species.

### Sample and RNA isolation for transcriptome sequencing

**Figure 1:**
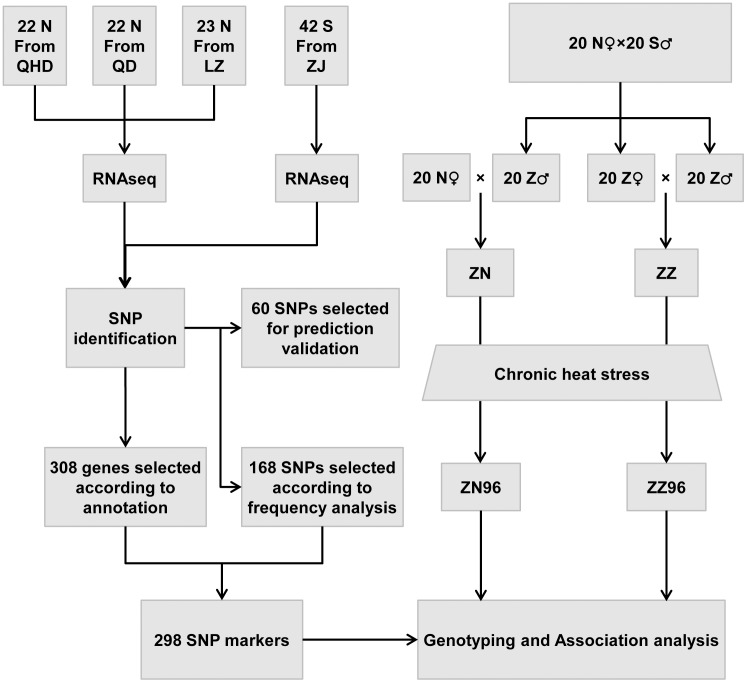
General flowchart for SNP identification and association analysis. Abbreviations: N, the northern subspecies of bay scallop; S, the southern subspecies of bay scallop; QHD, Qinhuangdao, Hebei Province, China; QD, Qingdao, Shandong Province, China; YT, Yantai, Shandong Province, China; ZJ, Zhanjiang, Guangdong Province, China; Z, hybrids of the two subspecies; ZZ, a descendant of the cross between Z (♂) and Z (♀); ZN, a descendant of the cross between Z (♂) and N (♀).

Sixty-seven northern bay scallop individuals from three different aquaculture populations (22 from Qingdao, Shandong province, 23 from Yantai, Shandong Province, and 22 from Qinhuangdao, Hebei Province) and 42 southern bay scallop individuals from Zhanjiang, Guangdong Province were used to make two pools for transcriptome sequencing (Fig. 1). For each subspecies, six tissues, i.e., the gill, mantle, adductor muscle, gonad, hemolymph and hepatopancreas, from each scallop were collected, pooled in equal weight and pestled in the presence of liquid nitrogen. The RNA was isolated from a fraction of the tissue sample using TRIzol reagent (Invitrogen) and following the manufacturer’s protocol.

### Library preparation for solexa sequencing

First, the RNA integrity was confirmed using an Agilent 2100 Bioanalyzer, and poly(A)+ RNA was enriched using a NEBNext Poly(A) mRNA Magnetic Isolation Module (NEB), followed by quantification using a Qubit Fluorometer (Invitrogen). For library preparation, 100 ng of poly(A)+ RNA was fragmented in 1× fragmentation buffer (Affymetrix) at 80°C for 4 min, purified and concentrated. Double-stranded cDNA was synthesized and end repaired, and a single ‘A’ nucleotide was added to the 3′ end of the blunt fragment before the index adapter was ligated (Illumina TruSeq RNA Sample Preparation Kit). Finally, the DNA fragment was enriched by PCR amplification and quantified by quantitative real-time PCR (KAPA Library Quantification Kit) before sequencing started on the Illumina Genome Analyzer II platform.

### *De novo* assembly

Raw reads with an average quality score less than 15 or that contained too many N (>5%) were removed, and any low sequencing quality bases at the 3′ end were trimmed. The *de novo* assembly of short reads was conducted using the Trinity software [39]. The transcripts were clustered by similarity using the BLAT [40] multiple sequence alignment tool, in which sequences with a perfect match of more than 80% of the longer transcript or 90% of the short transcript were represented by the longer one. In addition, a TGICL pipeline [41] was applied in which the sequences were first clustered based on their pairwise sequence similarity and then assembled by individual clusters to produce longer, more complete consensus sequences that produced the general unigene sequences of bay scallops.

### Gene annotation and differentially expressed genes

The general unigene sequences were blasted against the NCBI non-redundant (Nr) protein database and the Swiss-prot database using blastx with an E-value of 1E-5. In addition, both Gene Ontology analysis [42] and KEGG pathway analysis [43] were conducted.

The gene expression level was measured as reads per kilo-base per million reads (RPKM) according to Mortazavi et al. [44]. The IDEG6 web tool [45] was applied to identify differentially expressed genes (DEGs) between the two subspecies, and the results of all statistical tests were corrected by multiple testing with the Benjamini-Hochberg false discovery rate control (FDR <0.01). The sequences were deemed to be significantly differentially expressed if the adjusted *P* value was <0.01 and there was at least a two-fold change in RPKM between the two libraries. For enrichment analysis, a hypergeometric test was performed, followed by a Bonferroni adjustment. A significant enrichment was determined when the adjusted *P* value was <0.01.

### SNP calling and allele frequency analysis

To ensure the comparability of SNPs identified from the two subspecies, the general unigene sequences were used as reference for SNP identification. SNP calling was implemented for the two subspecies of bay scallops by four steps: (1) map clean reads back to the reference using the SOAP2 package with, at most, two mismatches allowed; (2) construct the consensus for each subspecies using the SOAPsnp package; (3) compare the consensus between the subspecies and call out both heterozygous and homologous SNPs; and (4) discard SOAPsnp calls if (a) the genotype score <30, (b) the sequencing depth ≤10 or ≥100, (c) for heterozygous locus, a sequencing depth of the minor allele <4 or the minor allele frequency (MAF) <5% and (d) more than 5 SNPs exist in the adjacent 50 bp. The SNPs from the two subspecies were classified into four categories (Figure S1): (1) the inter-subspecific SNPs derived between the northern and southern subspecies and the SNPs are homozygous in each subspecies; (2) the common SNPs shared by northern and southern subspecies and the SNPs are heterozygous in each of the subspecies; (3) the northern-subspecific SNPs, which are derived from only the northern subspecies and the southern subspecies has the same genotype as the reference; and (4) the southern-subspecific SNPs, which are derived from only the southern subspecies and the northern subspecies has the same genotype as the reference.

After filtration, 60 SNPs were randomly selected to validate the SNP prediction. Small amplicons, each containing one selected SNP, were obtained for eight individuals and genotyped using the high-resolution melting (HRM) method [46] (for detailed information, see the portion on “Association analysis based on heat tolerance related candidate genes”). The allelic imbalance score (the ratio between allele frequency of the northern and southern bay scallops) was calculated, and an allelic imbalanced SNP was determined when the score was ≥5 or ≤0.2 [47]. In addition, an enrichment analysis was conducted for unigenes containing subspecies-specific SNPs and imbalanced SNPs.

### Hybridization of the two subspecies and the heat stress experiment

To use intercrossed material for the association analysis, two populations were developed as follows: in 2011, northern bay scallops were crossed with southern bay scallops in a 20♀×20♂ manner according to Zheng et al. [48] to avoid self-fertilization. Briefly, each scallop was injected with 5-hydroxytryptamine (Sigma) to induce release of sperm. After sperm release (80–90 min), each spawner was rinsed thoroughly before egg releasing. Eggs were checked under microscope for sperm contamination, and the unexpectedly fertilized eggs were discarded. Then, eggs from 20 northern bay scallops were combined equally and fertilized by sperm from each of 20 southern bay scallops. In 2012, 20 hybrids were crossed with 20 other hybrids to generate a ZZ population or 20 northern bay scallops to generate a ZN population, using the same technique (♀×♂) as mentioned above (Fig. 1). To evaluate the heat tolerance of the two intercrossed populations, 40 northern bay scallops were mated in the same manner to generate an NN population. The three constructed populations were cultured in Jiaozhou Bay, China. Prior to the heat stress experiment, 100-day-old juvenile scallops from populations ZZ, ZN and NN were acclimated for two days at 25±0.5°C, in the laboratory in a 100-L tank with aerated, filtered seawater. For chronic heat stress, 233 individuals from the ZZ population, 230 individuals from the ZN population and 218 individuals from NN population were incubated at 26°C, and the temperature was gradually increased at a rate of 0.5°C per day. In this chronic heat stress experiment, the heat tolerance was measured as the accumulated heat stress (expressed as degree·hour, °C·hour) until death, assuming that the heat-susceptible scallops would die earlier and that heat-resistant scallops would die later. The mortality was monitored each day at 8 am, 3 pm and 9 pm, and dead scallops were collected for sampling. To compare the heat tolerance of the three populations, a one-way ANOVA was performed. The most heat-susceptible 48 individuals and the most heat-resistant 48 individuals were collected from both ZZ and ZN, denoted as ZZ96 and ZN96, and used in the subsequent candidate gene-based association analysis. The DNA of these scallops was prepared using a DNA extraction kit (Omega).

### Association analysis based on heat tolerance related candidate genes

Unigenes annotated as heat shock proteins, proteins involved in antioxidant activity (including superoxide dismutase, peroxidase, thioredoxin and glutathione redox system) and ubiquitin proteasome system (including ubiquitin, E1 ubiquitin-activating enzyme, E2 ubiquitin-conjugating enzyme and E3 ubiquitin ligase), together with unigenes containing allelic imbalanced SNPs, were selected as candidate genes for association analysis on heat tolerance. The following two types of SNPs located in these candidate genes could not be distinguished by the HRM method directly and were filtered out: (1) SNPs with a second SNP detected in the flanking 20 bp and (2) transversion between A and T and between G and C. After filtration, primers were designed with one SNP contained in each amplicon that ranged between 40 bp and 100 bp in length. For the amplification of the target SNPs, the DNA was diluted to 10–20 ng/µL, and 1 µL was used as template. PCR was performed in a 10 µL reaction volume at 94°C for 5 min, following 45 cycles of 94°C for 30 s, 55°C for 30 s and 72°C for 30 s using a PCR premix (DongSheng, China). The primer pairs were pre-detected using polyacrylamide gel electrophoresis, and only those that produced a unique and bright electrophoretic band that was consistent with the expected size were selected for genotyping in ZZ96. The SNPs that showed polymorphism in ZZ96 were further genotyped in ZN96.

Genotyping was conducted using a HRM method according to Wittwer et al. [46] with some modification. Internal temperature controls, i.e., complementary oligonucleotides that varied in G/C content and length, were used to shift and scale the temperature axis of the derivative melting plots, as described by Seipp et al. [49]. After the involvement of the internal temperature controls (1 µL at 10 µM for one reaction) and saturated fluorescent dye LCGreen (1 µL for one reaction), a small amplicon melting analysis was conducted on a LightScanner-96 instrument (Idaho Technology). The genotype of each SNP in each association population was collected, and the allele frequencies were compared between the heat-susceptible and heat-resistant groups using Fisher’s exact test in PLINK [50] v1.07.

### Quantitative expression analysis

Forty northern bay scallops were acclimated for one week at 23.5°C in a 100-L tank with aerated, filtered seawater in the laboratory. After acclimation, 20 individuals were subjected to two hours of heat stress at 30°C, and the 20 individuals that did not receive treatment were used as a control. Both the adductor muscle and gill were sampled for genomic DNA and RNA isolation, respectively. The genotype of each heat tolerance related SNPs for each individual was determined by HRM analysis. Then, individuals of each genotype were selected, and the expression pattern of the gene containing heat tolerance related SNP was investigated. Quantitative real-time PCR was conducted in a 20 µL reaction volume containing 0.4 µL of gene-specific primers (10 µM), 0.4 µL ROXII reference dye, 8.8 µL of diluted cDNA that was reverse transcribed from 1 µg of total RNA from gill tissue and 10 µL of TaKaRa Ex Taq™ SYBR premix. The mixture was subjected to the following conditions using an Applied Biosystems 7500 fast system: one cycle of 95°C for 30 s; 40 cycles of 95°C for 3 s and 60°C for 30 s; and one cycle of 95°C for 15 s, 60°C for 1 min and 95°C for 15 s. The primers used in this study are presented in Table 1. The relative expression was determined using the Livak 2^−ΔΔCT^ method [51].

**Table 1:** Primers used for qPCR to detect the heat tolerance-related SNP all-53308-760 T/C.

Index	Sequences*	Amplification efficiency**
all-53308 forward	ATGGATCTCAGAAGTGAACGT	0.998
all-53308 reverse	ATGATTCTAGCCTGTACCATTAC	
all-53308-T forward	GGATCTCAGAAGTGAACGTCTcA**T**	1.02
all-53308-C forward	GGATCTCAGAAGTGAACGTCTtA**C**	0.958
18S rRNA forward	CTGACCATAAACGATGCCGACT	0.938
18S rRNA reverse	AACTTTGGTTTCCCGTAAGCTG	

*The bases bold are an allele-specific base, and the bases in lowercase are a deliberate mismatch.

**The amplification efficiency of the allele-specific forward primer was determined in combination with all-53308 reverse primer.

For allele-specific quantitative expression analysis, two allele-specific primers were designed according to Ye et al. [52], with a deliberate mismatch encompassed at position −2 from the 3′-terminus to increase the specificity (Table 1). Then, in combination with the common forward or reverse primer, each allele-specific primer was used for the allele-specific quantitative real-time PCR analysis. To normalize the amplification efficiency of the two allele-specific primers, the genomic DNA of an individual that was heterozygous at the heat tolerance-related SNP was used for the allele-specific quantitative real-time PCR analysis. Because the genomic DNA contains equal amount of two different alleles, the difference in expression level of the alleles must be induced by different amplification efficiency of the two allele-specific primers. The coefficient of amplification efficiency of the two allele-specific primers was defined as 2^−ΔCT^, where ΔCT represents the difference of the CT value between two alleles. Then, the relative expression of each allele in the allele-specific quantitative real-time PCR was normalized according to the coefficient determined above.

### Statistical analysis

For the gene enrichment analysis, the results of the Gene Ontology and KEGG pathway analysis were further subjected to the hypergeometric test followed by Bonferroni adjustment [53], with the total gene list serving as the reference background. A significant enrichment was concluded when the adjusted *P* value was <0.01. The statistical significance of the differences in heat tolerance among populations and the expression level between alleles under different conditions were investigated using Student’s t-test, with a *P* value <0.05 considered statistically significant and a *P* value <0.01 considered highly significant. An association analysis was conducted using Fisher’s exact test in PLINK [50] v1.07. Both the missing data and the Hardy Weinberg Equilibrium were controlled (missing rate per SNP<0.5, missing rate per individual <0.1 and HWE >0.001), and individuals and SNPs that failed to meet the conditions were removed.

## Results

### Short read assembly

Two cDNA libraries were constructed from pooled RNA samples that were prepared from a total of 67 northern bay scallop individuals and 42 southern bay scallop individuals. The sequencing was performed using an Illumina Genome Analyzer II platform with 55.5 and 34.9 million 73-bp paired-end reads generated for the northern and southern bay scallops, respectively. After removing the low-quality reads, 46.9 million clean reads for the northern bay scallops and 29.4 million clean reads for the southern bay scallops were generated. The *de novo* assembly was conducted using clean reads from each subspecies and combined reads of both. The assembly using clean reads from the northern and southern subspecies produced 60,037 and 43,996 unigenes with an N50 of 654 bp and 497 bp, respectively. In addition, the assembly using combined clean reads from the two subspecies produced 71,909 unigenes with an N50 of 750 bp (Table 2). The sequence clustering was further conducted with unigene sequences of 454 sequencing (unpublished data) and produced 82,267 general unigenes with an N50 length of 834 bp.

**Table 2:** Summary of basic information after transcriptome sequencing.

Samples	No. of scallops	Clean reads (×10^6^)	No. of unigenes	N50 length
Northern	67	46.9	60,037	654
Southern	40	29.4	43,996	497
Total*	107	76.3	71,909	750
Clustered**	–	–	82,267	834

*Assembly using clean reads of the two subspecies combined.

**Unigene quantity and length information after clustering.

### Unigene annotation and differentially expressed genes

Among the 82,267 unigenes, 32,595 (∼39.6%) showed homology to at least one protein or domain in the Nr and Swiss-Prot databases, and 14,725 (∼17.9%) were assigned to at least one GO term. Furthermore, 9,270 unigenes were assigned pathway annotation when the KEGG pathway mapping was conducted (Table S1).

In total, 112 unigenes were down-regulated, and 43 were up-regulated in southern bay scallops (Table S2). Among the DEGs, 26 were annotated in the KEGG pathway database and significantly enriched in the extracellular matrix receptor interaction pathway (*P*<0.05), with one mucin-19-like gene up-regulated and three collagen genes down-regulated in the southern bay scallops. The GO enrichment analysis was also implemented, and enrichments in the peptidase inhibitor activity, structural molecule activity and ferric iron binding activity were observed (*P*<0.05).

### SNP calling and allele frequency analysis

**Figure 2:**
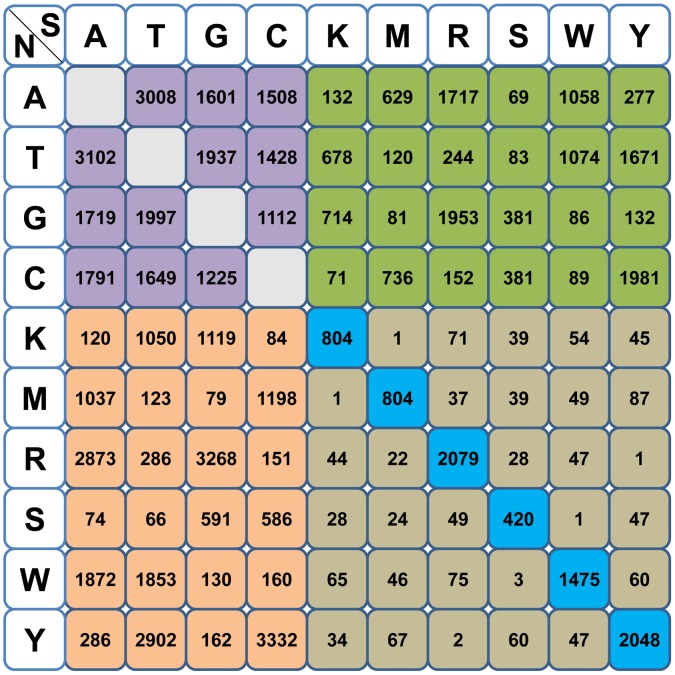
Classification and statistical analysis of SNPs in the two subspecies. A cell with a different background color represents different types of SNPs between the subspecies, and the number in each cell represents the amount of each type of SNP. Lilac: inter-subspecific SNPs, pink: northern subspecies specific SNPs, reseda: southern subspecies specific SNPs, azure: shared SNPs with the same heterozygosis type between the two subspecies and light brown: shared SNPs with a different heterozygosis type between the two subspecies. Abbreviations: N, the northern subspecies of bay scallop; S, the southern subspecies of bay scallop; A, Adenine; T, Thymine; G, Guanine; C, Cytosine; K, Thymine or Guanine; M, Adenine or Cytosine; R, Adenine or Guanine; S, Guanine or Cytosine; W, Adenine or Thymine; Y, Thymine or Cytosine.

SNP calling of the two subspecies of bay scallop was carried out by mapping filtered reads to the reference sequences, and SNPs were detected using SOAPsnp as described. The SOAPsnp calls were further filtered, generating a total of 32,206 and 23,312 high-quality SNPs for northern and southern bay scallops, respectively. Further, the SNPs of the two subspecies were compared and classified into four categories (Fig. 2): 22,077 were inter-subspecific between the two subspecies (Table S3), 8,803 were shared by the two subspecies (Table S4), 23,403 were specific to the northern bay scallop (Table S5) and 14,509 were specific to the southern bay scallop (Table S6).

Among the 8,803 SNPs shared by the two subspecies, a subset of 60 SNPs were chosen to validate the SNP prediction. Sixty pairs of primers were designed, and 36 were well amplified by pre-detection on PAGE gel prior to the HRM analysis (for the sequences of these primer pairs, see Table S7). Among the 36 well-amplified SNPs, 31 showed polymorphisms in eight randomly selected northern bay scallop individuals according to HRM analysis, indicating the high accuracy of the predicted SNPs. An allele frequency analysis of these shared SNPs was also conducted, and 261 SNPs showed striking differences in allele frequency between the subspecies (Table S8).

The KEGG pathway enrichment analysis was implemented for both northern- and southern-specific SNPs, and a significant enrichment in the oxidative phosphorylation and proteasome pathways was observed for the two datasets (Table S9). These two SNP datasets also showed significant enrichments in protein binding, endopeptidase activity and proton-transporting ATPase activity when GO enrichment analysis was performed (Table S10). However, there was no significant enrichment detected in the imbalanced SNP dataset.

### Do hybrids of the northern and southern subspecies possess a higher heat tolerance?

**Figure 3:**
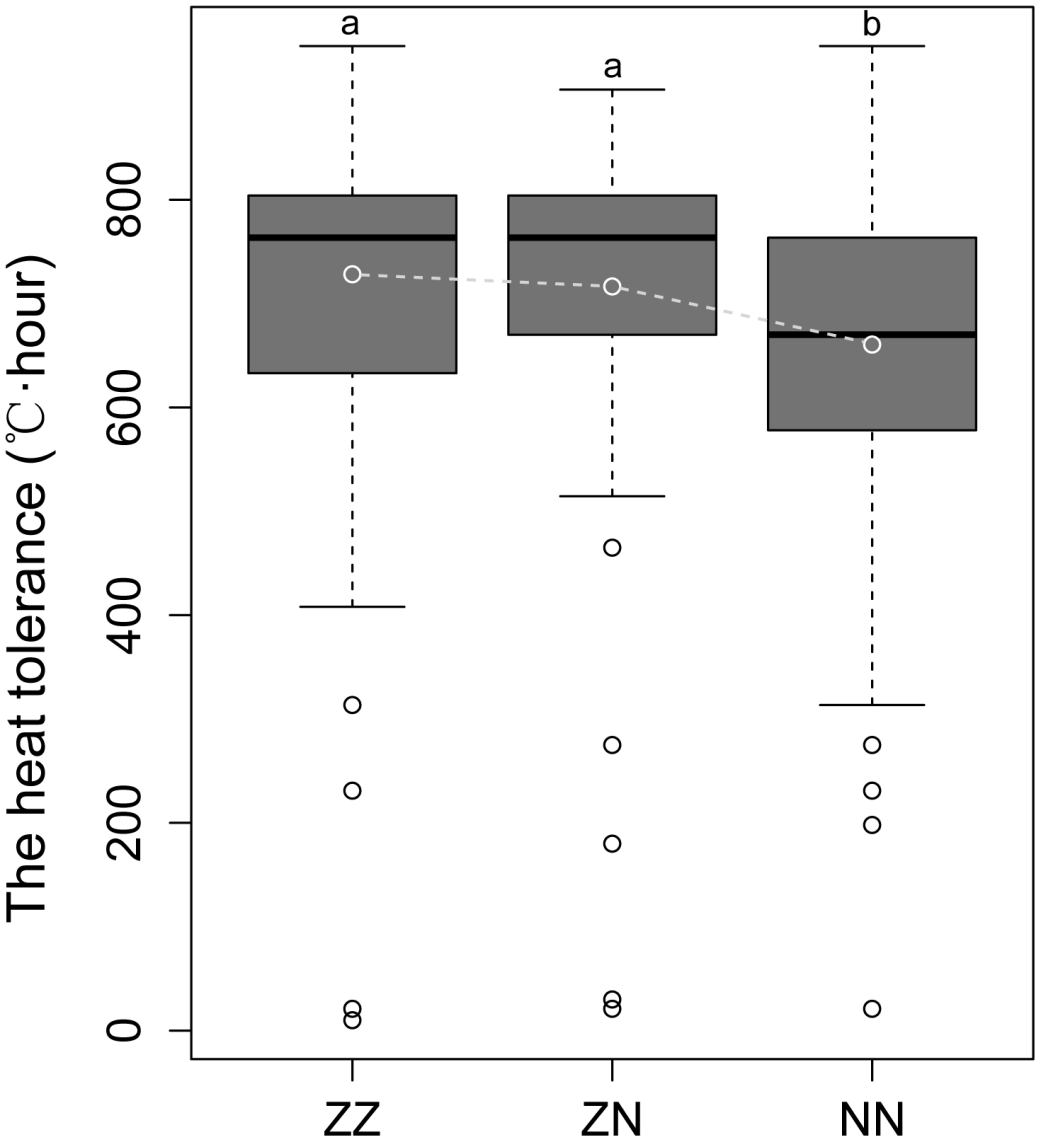
Comparison of the heat tolerance of populations ZZ, ZN and NN. The small white ball in each box represents the mean of the heat tolerance of the whole population. Different letters above the boxes represent a significant difference in heat tolerance between populations.

The individuals from the three acclimated populations ZZ, ZN and NN were subjected to chronic heat stress, and mortality was monitored three times each day. The heat tolerance of each individual in the three populations was recorded (Table S11). For the NN population, a sporadic death incident was observed before the accumulated heat stress reached 180°C·hour, after which point successive mortality occurred. However, for the two intercrossed populations of ZN and ZZ, successive death did not happen before the accumulated heat stress reached 465°C·hour, which was much higher than that of the northern bay scallop population NN. The mean values of heat tolerance for the three populations were compared, and the heat tolerance of the intercrossed populations, ZZ and ZN, were significantly higher than that of the northern bay scallop population NN (Fig. 3). ZZ96 and ZN96 each contained 48 of the most heat-susceptible scallops and 48 of the most heat-resistant scallops were used in the subsequent case-control association analysis.

### Association analysis on heat tolerance

In total, there were 115 unigenes annotated as heat shock proteins, 862 unigenes annotated as proteins involved in antioxidant activity (including 519 superoxide dismutase unigenes, 59 peroxidase unigenes, 155 thioredoxin unigenes and 129 glutathione redox system member unigenes) and 746 unigenes annotated as proteins involved in the ubiquitin proteasome system. These 1,723 unigenes, together with 241 unigenes containing allelic-imbalanced SNPs, were selected as heat tolerance candidate genes. Because the PCR amplicon-based HRM analysis would be adopted for genotyping, the SNP loci located in these genes were further screened, and 476 unigenes remained. Among these candidate genes, 308 unigenes were determined according to annotation information, and the other 168 unigenes contained imbalanced SNPs. Overall, 399 pairs of primers were designed, of which 298 amplified well (for the sequences of these primer pairs, see Table S7) and were further used for genotyping. Altogether, 136 SNPs showed polymorphism in ZZ96, of which 94 showed polymorphism in ZN96. Genotype data of these SNPs were collected, and a case-control analysis was conducted in ZZ96 and ZN96, respectively. Finally, 18 SNPs and 5 SNPs were determined to be related to the heat tolerance of bay scallops in ZZ96 and ZN96, respectively (Table 3). SNP all-53308-760 T/C was cross-validated in the two populations, and the T allele was advantageous to bay scallops for heat tolerance, while the C allele was adverse. In addition, there was considerable difference in the T allele frequency between the two sequenced natural populations (i.e. 0.583 in the northern and 1 in the southern subspecies, and thus it was determined to be a northern specific SNP). In total, 22 unigenes were determined to be related to heat tolerance of the bay scallop in at least one population. Among the 18 unigenes with a blastx hit, eight were related to protein homeostasis and the elimination of the ROSs. The others were involved in the molecular function of transcription activity, ion channel activity and signal transduction.

**Table 3:** Association results of two populations, assessed using Fisher’s exact test.

SNP	Type	Allele	Frequencies in ZZ96*		*P* value	Frequencies in ZN96*		*P* value
			susceptible	resistant		susceptible	resistant	
all-10291-285	C/T	C	0.3261	0.1023	0.0002709	–	–	–
all-12235-168	A/C	A	0.04444	0.1364	0.03771	–	–	–
all-14046-809	C/T	C	0.3605	0.3214	0.6297	0.4886	0.337	0.04886
all-2993-1987	G/A	G	0.131	0.3256	0.003294	0.2727	0.3953	0.1079
all-31130-1125	G/T	G	0.1548	0.3256	0.01182	0.2093	0.2667	0.3835
all-43668-953	C/T	C	0.2143	0.3953	0.01259	–	–	–
all-43684-1766	C/A	C	0.5652	0.3488	0.004372	0.4432	0.5319	0.2397
all-4912-525	G/A	G	0.2826	0.4535	0.02012	0.2609	0.383	0.08561
all-4989-3302	C/T	C	0.4643	0.4186	0.6433	0.4205	0.2444	0.01671
**all-53308-760**	C/T	C	0.3404	0.1064	0.0001847	0.3295	0.106	0.0002715
n-10658-1017	A/G	A	0.2308	0.4651	0.001936	0.2111	0.234	0.7267
n-11978-1212	T/C	T	0.381	0.186	0.006229	–	–	–
n-13362-1534	A/G	A	0.4103	0.1786	0.001681	–	–	–
n-17218-1253	T/C	T	0.2317	0.4359	0.00728	0.3864	0.4556	0.3664
n-30227-1794	A/G	A	0.5341	0.314	0.003723	0.413	0.3936	0.8813
n-37639-1689	G/T	G	0.1585	0.02381	0.002537	–	–	–
n-53835-1616	A/G	A	0.2619	0.5119	0.001443	–	–	–
n-63402-535	C/T	C	0.4205	0.3488	0.3534	0.5	0.2447	0.0004227
n-63715-817	A/G	A	0.025	0.1395	0.01024	0.06667	0.06383	1
n-63953-874	G/A	G	0.2	0.3864	0.01103	0.1932	0.2717	0.2233
n-8980-801	T/C	T	0.2065	0.25	0.5896	0.2	0.337	0.04516
s-8230-781	A/C	A	0.025	0.1136	0.03445	–	–	–

*Please see the “Hybridization of the two subspecies and the heat stress experiment” section for ZZ96 and ZN96.

### Expression variation of Gene all-53308 is related to the heat tolerance of the bay scallop

Based on the case-control analysis above, it was speculated that all-53308 may function as a node in the gene network related to the heat tolerance of the bay scallop, and thus that a change in its expression or protein structure may affect the scallop’s heat tolerance. Because it was an unknown gene, we first detected whether the expression variation exists among individuals with different genotypes under both control and heat stress conditions.

**Figure 4:**
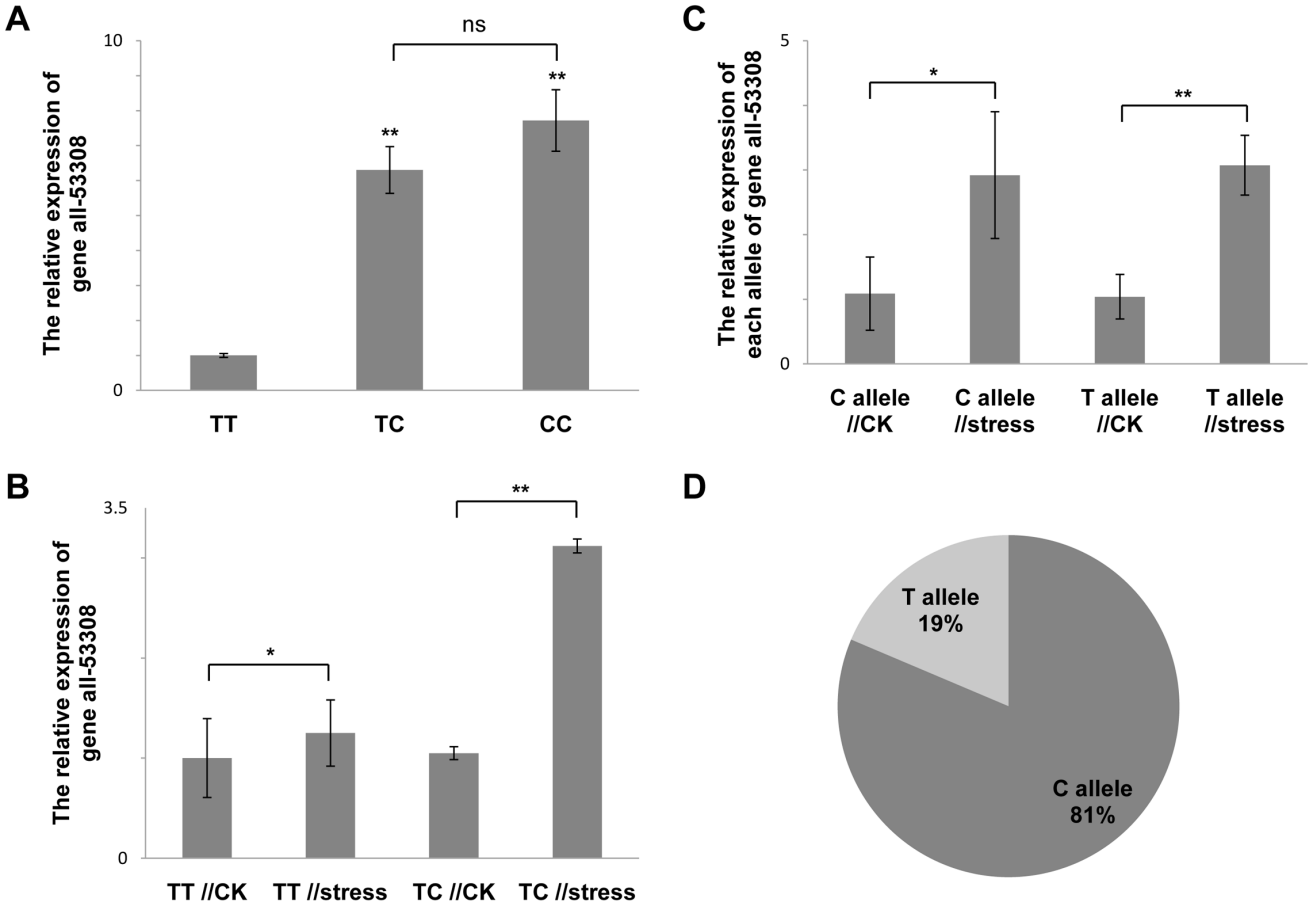
Quantitative real-time PCR analysis of gene all-53308 under both control and heat stress conditions. A) relative all-53308 mRNA expression in individuals with the TT, TC and CC genotypes under the brachychronic heat stress conditions; B) all-53308 mRNA expression in individuals with the TT and TC genotype under normal and brachychronic heat stress conditions; C) relative allele-specific all-53308 mRNA expression in individuals with the TC genotype under normal and brachychronic heat stress conditions; D) relative allele expression in individuals with the TC genotype under brachychronic heat stress conditions. The values are displayed as the mean ± SD of triplicate independent experiments. Differences that were determined to be statistically significant are indicated by asterisks (*P<0.05 and **P<0.01); ns, not significant.

To begin, we demonstrated that all of the primer pairs used in this study had a good amplification efficiency (Table 1). Then, 20 heat shock-treated individuals (16 individuals with the TT genotype and 4 individuals with the TC genotype) and 20 controls (12 individuals with the TT genotype, 5 individuals with the TC genotype and 3 individuals with the CC genotype) were collected for quantitative expression analysis. Different gene expression levels were observed in individuals with different genotypes under both heat stress and control conditions (Fig. 4A and Fig. S2). Two hours of brachychronic heat stress led to a significant up-regulation of gene expression in individuals with the TC (*P*<0.01) and TT (*P*<0.05) genotypes (Fig. 4B). However, this increased range of gene expression was much steeper in individuals with the TC genotype than in individuals with the TT genotype under the heat stress conditions (Fig. 4B). Because allele T was favorable to heat tolerance in bay scallops, the gene expression level of all-53308 was thought to be negatively correlated with the heat tolerance of the bay scallop.

To explore the gene expression pattern of each allele of the heterozygous individuals, allele-specific quantitative real-time PCR was conducted. A significant increase in gene expression was detected in both the T and C alleles under the heat stress conditions (*P*<0.05, Fig. 4C). However, when the expression level of the T allele of the heterozygous individual was compared with that of the C allele, a much higher expression level was observed in the C allele than in the T allele. To normalize the relative expression levels of the two alleles, quantitative real-time PCR using genomic DNA of the individual as a template was performed. The calculated coefficient of amplification efficiency between the C and T alleles was 1.11, and the normalized ratio of the expression level was 4.37 to 1 (Fig. 4D) and 4.92 to 1 (Fig. S3) under the heat stress and control conditions, respectively.

## Discussion

Prior to this study, two EST databases had been developed for bay scallops by Roberts et al. [54] and Song et al. [55], who provided 137 and 2,779 unique sequences, respectively, that served as a very important resources for gene clone [56], [57] and marker development [58], [59]. However, the available sequence resources are not sufficient for linkage mapping or association analysis, which are common and effective approaches for the genetic architecture dissection of complex traits. Because the sequencing cost decreased dramatically with the advent of the next-generation sequencing, it is quite cost-effective to develop genome-level genic SNPs using transcriptome sequencing.

In this study, RNA-seq using pooled RNA samples from multi-individuals was conducted for both northern and southern bay scallops using the Illumina Genome Analyzer II platform. The *de novo* assembly was implemented, and 82,267 unigene sequences were obtained, of which 31,681 genes were annotated, providing the most comprehensive packet of sequences for the bay scallop to date. There were 155 genes differentially expressed between the subspecies. Significant enrichments in the extracellular matrix receptor interaction pathway, peptidase inhibitor activity, structural molecule activity and ferric iron binding activity were detected. Because the southern bay scallop was brought from Zhanjiang to Qingdao and temporarily reared in different conditions compared with northern bay scallops before sample collection and RNA isolation, these enriched DEGs may reflect the different effects of environmental change (including temperature, salinity and microflora) and the sampling conditions for the two subspecies.

In total, 32,206 and 23,312 high-quality SNPs were identified in northern and southern bay scallops, respectively. This result indicates that the two aquaculture stocks of bay scallop in China still possess high genetic diversity after nearly 30 years of farming. This diversity may benefit from the group breeding and spawning practices used in the industry, which incorporate hundreds to thousands of bay scallops to supply eggs and sperm [60]. Additionally, such a high genetic diversity could serve as a guarantee of the long-term sustainable aquaculture of bay scallops in China.

The SNPs of the two subspecies were further classified into four categories, i.e., northern subspecies-specific, southern subspecies-specific, northern and southern subspecies shared and inter-specific. Sixty pairs of primers were designed for the SNP prediction validation from the shared SNPs. Among the 36 pairs of primers that were well amplified, 31 were polymorphic in eight scallop individuals that were collected at random. It was determined through this verification that over 86% of the predicted SNPs were correct. Furthermore, it was proposed that more loci would have been polymorphic if more individuals had been used. These predicted SNPs, including the 329 that were validated (including 31 randomly selected for validation and 298 from candidate genes for association analysis), could be valuable marker resources for linkage mapping and association mapping in future research.

Enrichment analysis was conducted for both northern- and southern-specific SNPs. Significant enrichment was detected in oxidative phosphorylation and proteasome pathways and in protein binding, endopeptidase activity and proton-transporting ATPase activity. This result implies that the genomic structural divergence in the genes related to energy metabolism and defense may be involved in the adaptation to the different temperatures for the bay scallops.

According to the chronic heat stress experiment, the ZZ and ZN populations were more heat tolerant compared with the NN population. It is suspected that the involvement of the germplasm from the southern bay scallop is responsible for the significantly improved heat tolerance of the two intercrossed populations. This finding is consistent with the recognition that the southern bay scallop is naturally distributed in a region of relatively higher temperature and should thus be more heat tolerant than the northern bay scallop.

The allele frequencies derived from RNA-seq may not precisely reflect the authentic allele frequencies in the population due to allele-specific expression [61]–[64]. Schunter et al. [65] suggested that using 12 individuals for RNA-seq provided a nearly accurate prediction of the true allele frequencies in populations. Thus, sequencing on over 40 individuals in each subspecies was intended to provide a credible estimation of the allele frequencies for both sequenced natural populations of bay scallop. The SNPs that show conspicuously different allele frequencies between the subspecies were conjectured to be responsible for their different heat tolerances. Hence, an allelic imbalance analysis was conducted, and a striking allele frequency difference was detected in 261 high-quality SNPs.

Consequently, in this study, unigenes that annotated as heat shock proteins, proteins that were involved in antioxidant activity and ubiquitin-proteasome system, together with unigenes that contained allelic imbalanced SNPs, were selected as candidate genes for the association analysis on heat tolerance. In total, 22 SNPs were detected to be related to the heat tolerance of the bay scallop in at least one association population. Among the unigenes involved, 18 had a blastx hit in the Nr database. Eight of these genes were related to protein homeostasis and the elimination of ROSs, and the others were involved in the molecular function of transcription activity, ion channel activity and signal transduction. SNP all-53308-760 T/C was cross-validated to be closely related to the heat tolerance of the bay scallop in the two association populations. Notably, the two sequenced natural populations showed a considerable difference in T allele frequency. It is implied that the SNP all-53308-760 T/C may have been subjected to natural selection for temperature adaptation and that the higher frequency of T allele in the southern subspecies is the consequence of local adaptation. However, it is unclear whether there is also a significant difference in allele frequency at this locus in the wild populations of the two subspecies along the eastern cost of America.

It was conjectured that SNP all-53308-760 T/C or a haplotype containing this locus might indirectly influence the heat tolerance of the bay scallop by altering gene expression. Quantitative expression analysis was conducted, and a significant difference in expression was detected between individuals with different genotypes under both control and heat stress conditions. This difference may indicate the existence of a different expression pattern between the T and C alleles. Thus, allele-specific quantitative real-time PCR was further implemented in heterozygote individuals, in which the expression pattern of a particular allele was determined. A striking difference in expression level was observed between the C and T alleles, and the normalized ratio of expression level was over four to one. In combination with the association analysis, we propose that the gene expression level of all-53308 was negatively correlated with the heat tolerance of the bay scallop.

The quantitative expression analysis also revealed a different expression pattern of the T allele between the TT and TC genotype individuals. It is speculated that the C allele is in high linkage disequilibrium or on the same haplotype with another variation that regulates gene expression of all-53308 in trans and most likely does so in a dominant manner. Though further study is required to address the role of all-53308 in the heat tolerance of bay scallops, our results demonstrate that SNP all-53308-760 T/C may serve as a marker for breeding practice.

## Supporting Information

Figure S1 Principles for the classification of SNPs between the subspecies.
(TIF)

Figure S2 Relative expression of all-53308 in individuals with different genotypes under control conditions.
(TIF)

Figure S3 The normalized ratio of the expression level between alleles under control conditions.
(TIF)

Table S1 Annotation information.
(XLSX)

Table S2 Differentially expressed genes and annotations.
(XLSX)

Table S3 List of inter-specific SNPs.
(XLSX)

Table S4 List of shared SNPs.
(XLSX)

Table S5 List of northern bay scallop-specific SNPs.
(XLSX)

Table S6 List of southern bay scallop-specific SNPs.
(XLSX)

Table S7 List of primer pairs that amplify well.
(XLSX)

Table S8 Imbalanced SNPs between the subspecies.
(XLSX)

Table S9 KEGG enrichment analysis information of subspecies-specific SNPs.
(XLSX)

Table S10 GO enrichment analysis information of subspecies-specific SNPs.
(XLSX)

Table S11 Heat tolerance of the ZZ, ZN and NN populations.
(XLSX)
