# Supplementary figures and images for: SNP Identification by Transcriptome Sequencing and Candidate Gene-Based Association Analysis for Heat Tolerance in the Bay Scallop Argopecten irradians
Source: PLoS One. 2014 Aug 14;9(8):e104960. doi: 10.1371/journal.pone.0104960 (PMC4133247; doi:10.1371/journal.pone.0104960)

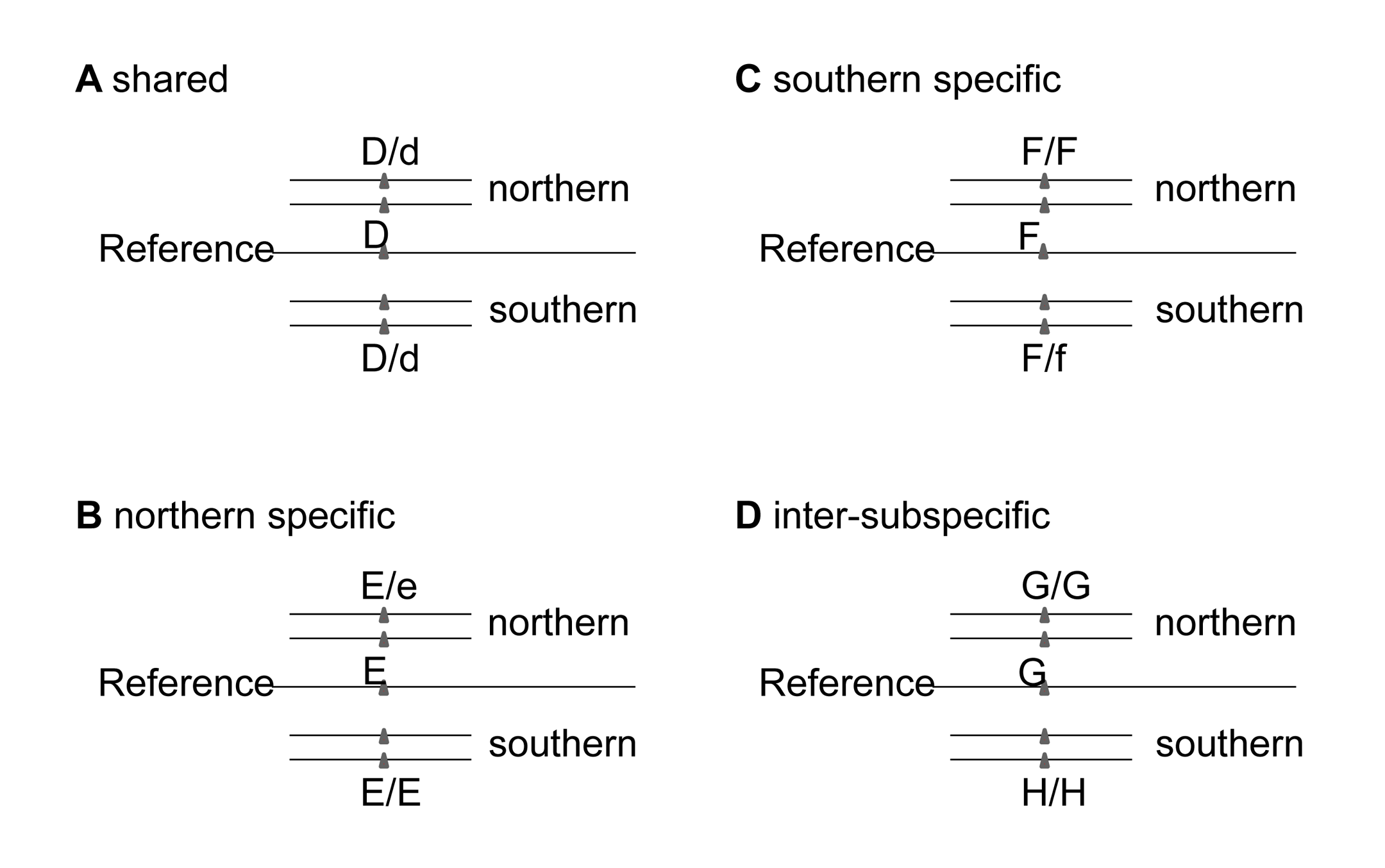

Supplement: Figure S1 — Principles for the classification of SNPs between the subspecies. (TIF) [file pone.0104960.s001.tif]

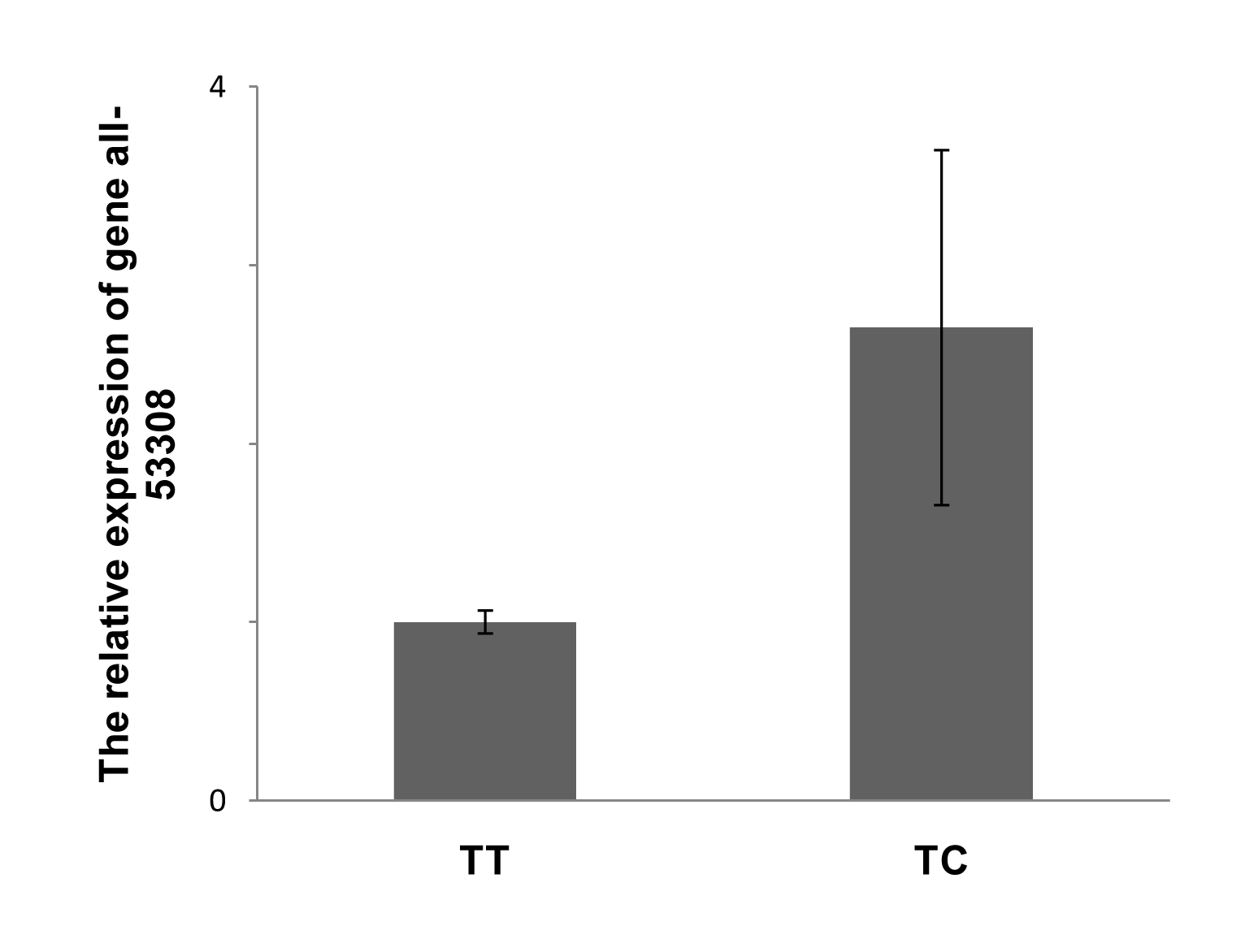

Supplement: Figure S2 — Relative expression of all-53308 in individuals with different genotypes under control conditions. (TIF) [file pone.0104960.s002.tif]

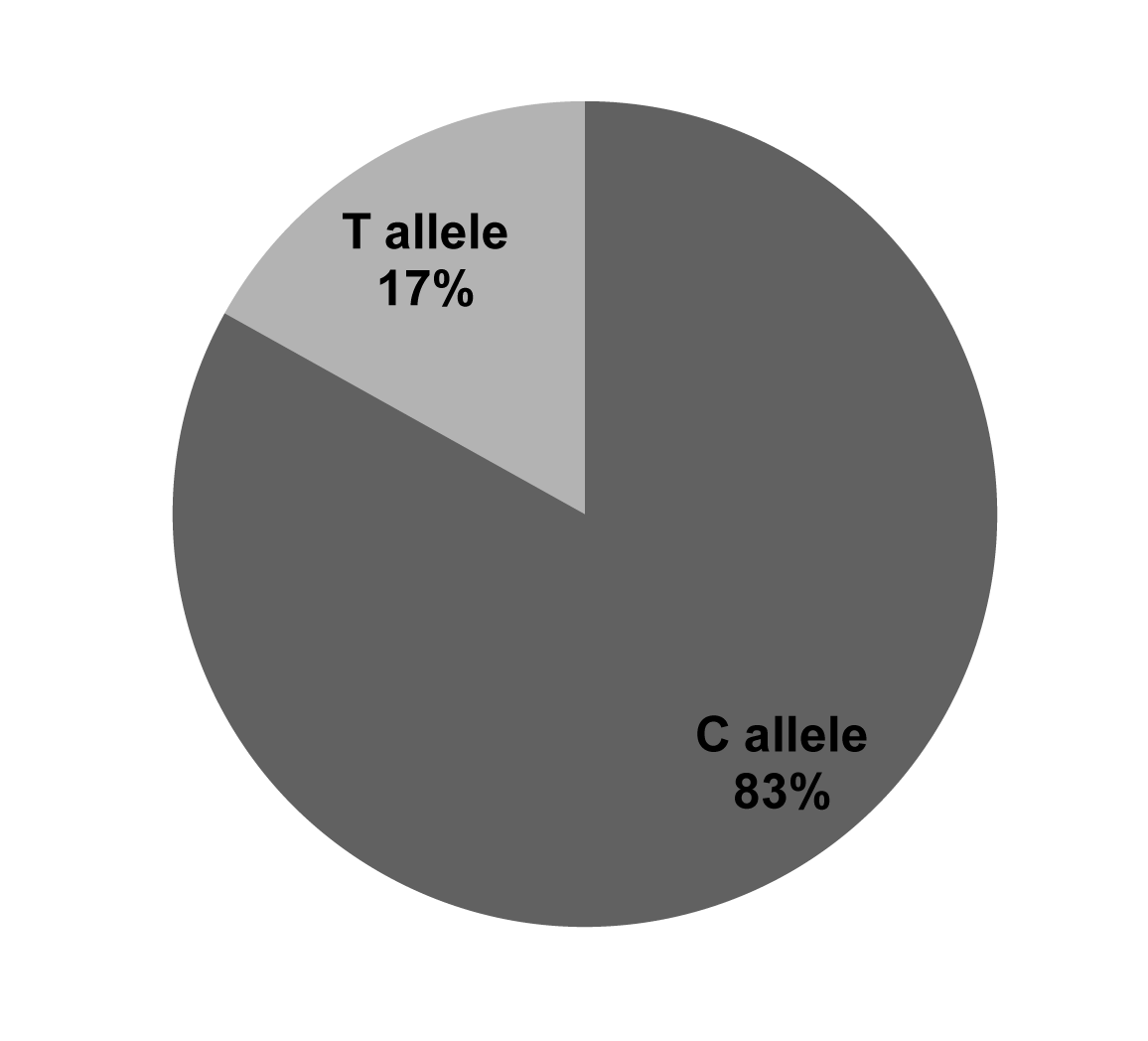

Supplement: Figure S3 — The normalized ratio of the expression level between alleles under control conditions. (TIF) [file pone.0104960.s003.tif]
